# Unusual Advice to Prevent Burnout in Early Medical career

**DOI:** 10.15694/mep.2020.000030.1

**Published:** 2020-02-13

**Authors:** Vijay Rajput, Daniel Hutchinson, Anuradha Lele Mookerjee

**Affiliations:** 1 Nova Southeastern University; 2 Cooper University Hosptial; 3 Cooper Medical School of Rowan University

**Keywords:** Career Advice, Well-being, Mentoring

## Abstract

This article was migrated. The article was marked as recommended.

Burnout is known to be a chronic response to prolonged occupational stressors. Learners, faculty, and other health care professionals experience burnout since they often lack the emotional, physical, and mental means to overcome the demands of professional work, their careers and life in general. Furthermore, higher demand for patient care revenue, teaching, scholarship and research has increased the prevalence of psychological strain and burnout among physicians. Every health care professional need guidance when starting a new job and career after the training period. A medical student’s goals differ from those of a post graduate trainee (resident or fellow) and a resident’s current goals will differ from those of his or her first “real” job. This article will explore simple advice to young doctors who are embarking on their first job post residency training. It will explain the importance of valuable mentorship, how to become a regional expert and how to juggle responsibilities in the workplace and home. It highlights the importance of writing and journaling reflections and preparing for unexpected twists during a medical career and why all physicians should strive to make time for themselves and their hobbies. Inculcating these simple habits and attitudes can make the physician’s job more rewarding and meaningful. Physicians following these tips and guidelines may find more fulfilment and meaning in their professional and personal lives leading to lesser burnout on the job.

## Introduction

Occupational stress and exhaustion have a negative impact on one’s professional life leading to poor quality work, low morale, absenteeism, and decreased motivation. Health care professionals’ burnout has a direct impact on professionalism in medical education and patient care. The transition from the safety net of training to the independent career can be intimidating. The difference between a health care professional’s new job search and other later job searches is two-fold. First, the new position comes with challenges such as malpractice insurance, state licensing, staff privileges, legal contracts, and salary negotiations. These topics can dominate residents’ checklist of things to do before starting the new jobs. Second, health care professionals’ sources for growth and learning will change dramatically after clinical training. The students meet the practice type they choose and the interests they pursue will all shape their development as young physicians. Although each health care professional’s career goals will differ, an understanding of these simple, but often overlooked, principles will help residents achieve meaningful careers.

## 1. Expertise/Mastery is More Important than Money in Early Career (Get paid to learn)

The sharp overnight increase in a resident’s monetary gain can feel seductive. This temptation will intensify with a quick calculation of how the extra dollars/ amount / monies will expedite debt payments. Residents should have role models who can help develop good habits and stay away from frugal lifestyles. They should evaluate employers not by their financial offerings but by how well they can foster the individual growth and development. Young professionals should identify colleagues and seniors who are skilled and knowledgeable and who have shown expertise in clinical areas so that they can refer their patients with confidence. With increasing autonomy in their practice, they should cultivate new habits and routines.

## 2. Become a Local or Regional Expert on One Unique knowledge or point of view

Expertise results from a combination of education and experience. Until now, training programs were a source for both. When residents begin practice, their experience will continue to accrue while their education becomes their own responsibility. At the start, these residents should choose a single disease or procedure in their respective practice. They should build a folder on their computer and keep track of every new study or article related to that topic. Furthermore, they should develop a true expertise in this area and become a local expert for educating their peers. These efforts will help establish their reputation in their communities and help them receive referrals for their field of expertise. This proficiency can expand into regional and national expertise and open opportunities for research and scholarship. This also helps in increasing engagement with colleagues and healthcare professional and prevents burnout.

## 3. Have Majors and Minors in A Professional Career

In U.S., colleges’ undergraduate students have a “Major” and “Minor,” or two areas of study in which they focus. This concept, when applied to physicians, can provide the opportunity to explore different interests in a career. The “major” refers to a specialty practice while the “minor” can be an area of patient care or system improvement. This differs from the typical advice for developing an expertise since the second area of interest is not a topic one would encounter in the “major.” Instead, the “minor” interest is a distinct area of professional development. Examples of these interests include quality, safety, medical ethics, legal, risk management, administration. Ultimately, development of these interests can provide an outlet for shifting professional focus later in the career. This shift may be more transformative for physicians who want to alter and transition to a non-clinical practice. As young physicians start to practice, they will begin to discover the “minor” and append it to their “major” which can lead to a conversion of their future career. The change of direction with many of the minor interests can lead to a new career path which can ultimately prevent exhaustion and burnout on a long-term trajectory.

## 4. Be Surrounded by Learners

Physicians should attempt to surround themselves with outstanding and enquiring learners, who can provide both teaching and learning opportunities. Most residents during their training period undergo rigorous teaching and educational activities which strengthens their clinical expertise. In practice, learners provide residents the same intellectual stimulation, while also providing a platform for reflection that is crucial for maintaining academic competence and professional engagement ( Williams, 2001). The questions posed by students and residents can help practicing physicians to observe and reflect on the disparity between learning and professional practice in real time.

## 5. Walk Before Running (Effectiveness Before Efficiency)

In a current volume-based reimbursement system, payers reward efficiency. Becoming an efficient clinician is certainly a valuable skill. The road to efficiency, however, can be traveled either through experience or through haste. To enhance efficiency, physicians are forced into making ‘short cuts’ and become “cognitive misers.” Most Individuals have fixed cognitive capacity to process and integrate information. According to the social cognition theory, when a learner is faced with excessive cognitive load, they will apply the automatic pattern recognition process or heuristic approach to their tasks. These cognitive stingy approaches tend to occur to maintain a sense of efficiency during their increased cognitive load ( Fiske, 1984). In the initial years after training, this hasty style of practice will be tempting, but one should resist it. The new practitioner should think about their role models during their training because these clinicians were systematic and methodical and went the extra mile for their patients. It is in the new physicians’ best interest to emulate these qualities and be effective from early on.

## 6. Juggle Work and Life – Don’t Try to Balance It

There is evidence to suggest that health care professionals and other related faculty who focus on the work that is meaningful to them and maintain a career/life harmony have a lower risk of burnout. Healthier lifestyle choices, career acceptance, and collegial support are some of the many methods to prevent burnout development. This Zen-inspired phrase of work-life balance is a poor description of real life. There is no true balance of work and life that one can achieve. A mentor once said- a person has three crystal balls: work, family and integrity. He or she can only hold two at a time, one in each hand. Since this person can never break his or her integrity, one hand will always be occupied by the ball of integrity. This person’s task then, is to find a way to continually juggle work and family in the other hand. He or she will of course, never neglect work or family completely. There will be times, however, when one demands more of their attention requiring a shift away from the other. It is during these times he or she will throw the other ball a little higher, suspending it for a little longer as the focus shifts to that ball. It is important to be careful, since the higher one tosses the crystal ball in the air, the more one risks the chance of breaking it.

## 7. Apply 80/20 Rule for Work and Relationships

When entering practice, the 80/20 rule can be a yardstick to measure how well a resident fit in with his or her current job position. Although there are many iterations of the 80/20 rule in business world ( Koch, 1998). In this instance it means that a resident’s evaluation of a job or relationship is 80% positive and 20% negative. Residents should then ask themselves these questions: Do they find themselves engaged and fulfilled at least 80% of the time? Are they happy four out of five days during the job? Do they feel valued by superiors 80% of the time at work? Are the feelings of disorganization, overwhelming responsibility and melancholy limited to 20% of the time? If they answered yes to these questions, they have found a position where they fit well. In their personal life, residents should apply this same rule to their significant others, kids and/or family members. It is important to keep in mind there is no perfect match or an ideal job. The level of acceptable discomfort in any situation can be set reasonably at 20 percent. If it begins to consistently rise above this, residents should recognize their discomfort and make changes to improve it.

## 8. Find Mentors and Mentees both within and outside of the Professional Career

Mentoring is about making a true difference in mentees’ lives and helping them learn how to think for themselves ( Daloz, 1999). Mentors can be local, regional or global. These relationships can be formed in the profession or outside of the field. Residents may find mentors who help in specific parts of their lives or those that help them through many difficult life decisions (changing jobs, having kids, moving to another state). There is no right number or type of mentors. The mentor-mentee relationship is a dynamic process that changes both the resident and the mentor as they both propagate and flourish in each other’s life story. They both grow in their lives with mutual benefits.

At the same time, these young physicians should become mentors to younger trainees and help disseminate the ideals that they have learned from their experiences. The relationship they choose to develop as both a mentor and mentee is more than just a professional network. They are a source of perspective and challenge to the young trainees’ visions of their careers. They can also provide a safe space to reflect on difficulties in their lives and how to overcome them. With a mentor or mentee, a phone call on the weekend or a chat over coffee can be as refreshing and rejuvenating like a vacation and give relief from the stressors on the job.

## 9. Reflect through journaling and reading about experiences

During residency training it can be hard to find time to write outside of patient care communication, but one needs to carve out ten minutes daily and write about the complex and challenging patient situations and reflect on the experience.They should not worry about the language and syntax; they should just write. They can find time later and go back and refine the writing. They should also be sure to write down new ideas, connections they made between diseases, or diagnoses that they saw for the first time. The benefit of this journaling is the actual processing of the emotions experienced during the day during a patient interaction. In addition to taking a few minutes to journal, residents should also try to read something outside of medicine. When reading they should develop the practice of taking notes and extending the relevant principles and apply it to their daily practice. Over the years, the notes formulated from the reading can help create frameworks or philosophies that are applicable to their practice, teaching and mentoring.

## 10. Be Ready for Unexpected Twists in a Professional Career

Any given resident has spent thousands of dollars and hours preparing for a career as a doctor. In the unlikely event an accident impairs a resident’s ability to work as a healthcare professional, it may take some time for him or her to find a new position. Additionally, residents’ financial earning potential can drop significantly, so it is important that they are prepared and always buy right disability insurance coverage. The last important piece of advice for residents is for them to always carry their medical licenses from two nearby states. This is helpful if the health system has clinics in nearby states or a resident decides to move to a nearby state without moving with their entire family.

## Conclusion

The advent of burnout has been on a progressive rise among physicians due to individual and organizational factors. Due to the increasing prevalence of burnout, interventions to combat burnout are being researched across the nation. According to a recent systematic review and meta-analysis, some useful interventions include reducing duty hours, learning about mindfulness practice and stress management, as well as being involved in small group discussions. Although there are many interventions to overcome burnout, the prognosis is uncertain from one individual to another.

Physicians should take the initiatives outlined above and need to recognize what promotes joy, effectiveness and engagement, with the view to improving work and satisfaction. It is also important that physicians be aware of the various ways to combat burnout to improve work performance, motivation, student learning, and patient safety. The ten guiding principles outlined above can help to achieve a meaningful and fulfilling personal and professional life.

## Take Home Messages

• It is more important for young health care professionals in their first new job to focus on building and refining skills, rather than concentrate on the monetary value that the job gives.
• Develop deep and meaningful relationships with mentors and mentee both within and outside of medicine to decrease emotional exhaustion.
• Find time for reflection including journal writing and reading.
• The 80/20 rule should be used to decide whether the current job is a good fit for them.
• Develop a major and minor in your area of expertise to build a meaningful and fulfilling career and prevent burnout.

## Notes On Contributors

Dr Vijay Rajput, MD is a Professor and Department Chair of Medical Education at Nova Southeastern University, Dr Kiran C. Patel College of Allopathic Medicine, Fort Lauderdale, Florida, USA.

Dr Daniel Hutchinson, is a first year Internal Medicine resident at Cooper University Hospital at Camden New Jersey, USA.

Dr Anuradha Lele Mookerjee, is an Associate Professor of Medicine and course director at Cooper Medical School of Rowan University, Camden, New Jersey, USA.
