# Supplementary material for: Unusual Advice to Prevent Burnout in Early Medical career
Source: MedEdPublish (2016). 2020 Feb 13;9:30. [Version 1] doi: 10.15694/mep.2020.000030.1 (PMC10697501; doi:10.15694/mep.2020.000030.1)
Supplement: Peer review history

# Reviewer report: Reviewer response for version 1

**DOI:** 10.21956/mep.19976.r30375

**Published:** 2020-04-06

**Authors:** Helena Filipe

**Affiliations:** 1 Hospital of the Armed Forces/PL-EMGFA

This review has been migrated. The reviewer awarded 5 stars out of 5 Thank you for the opportunity to review this article.This is a well written perspective focusing on good mentorship as essential to develop professionalism and avoid burnout, while a young practicing physician. Despite my biased personal attraction for the topic, mentorship still remains a key learning method in the medical education spectrum, so evidence as well informs. Healthcare professionals find job satisfaction in achieving good patient outcomes and in feeling their work regarded as a contribution for public good. Keeping own personal well-being and feeling engaged in work is essential for those achievements.I concur with the point of view synthesised by the Authors and acknowledge their good collection of useful and practical principles to minimize the risk of burnout. Globally spreading across several healthcare cadres, burnout calls out attention for the imperative need of healthcare professionals to look after themselves to better take care of others.Since ancient scripts, mentorship is presented as the traditional and effective learning method throughout medical education history. This article pulls mentorship forward as a good approach to reduce the risk of burnout.More than ever, identifying good role models and finding good mentorship are key to become a fulfilled healthcare professional, such are the challenges, setbacks and allures posed by the increasing complex healthcare systems. I would have welcome these guidelines in my early days as a physician, will surely hold them as very objective direction to me and to inspire and guide colleagues to find work enjoyable, well regarded, worth to invest energy and dedication, find the wonders of serving others, and making a difference. A gem to bring close.

Reviewer Expertise:

NA

No decision status is available

# Reviewer report: Reviewer response for version 1

**DOI:** 10.21956/mep.19976.r30377

**Published:** 2020-02-28

**Authors:** P Ravi Shankar

**Affiliations:** 1 American International Medical University

This review has been migrated. The reviewer awarded 4 stars out of 5 This is an interesting paper on burnout and career advice to young physicians. The paper is well-written and is an enjoyable read. The point about juggling three balls at the same time – integrity, career and family is interesting. There is a lot of pressure on doctors and educators to compromise on integrity from different sources. I liked the analogy of throwing the ball higher and allowing it to remain in the air longer when we have to put something on the back burner for the time being. The advice of developing an area of specialization in one’s career and of interests outside medicine is interesting. The 80:20 rule is also very true as there is no job or situation with which you can be happy all the time. All educators and young doctors beginning their careers will find this paper to be of interest. Some of the advice is unusual, others less so but all seem to be very relevant.

Reviewer Expertise:

NA

No decision status is available

# Reviewer report: Reviewer response for version 1

**DOI:** 10.21956/mep.19976.r30379

**Published:** 2020-02-24

**Authors:** BALAJI ARUMUGAM

**Affiliations:** 1 TAGORE MEDICAL COLLEGE AND HOSPITAL

This review has been migrated. The reviewer awarded 4 stars out of 5 Thanks for the invite to review the article. Its a great pleasure to review this personal opinion article on burn out among physicians and the proposed interventions. Learners, faculty, and other health care professionals experience burnout since they often lack the emotional, physical, and mental means to overcome the demands of professional work, their careers and life in general. I completely agree with this sentence which states clearly the lack of means to overcome. The best part of this opinion article which I personally liked is the Expertise is more important than money in early career and the walk before running i.e effectiveness before efficiency but it could have been better explained. Doing the right things and master on this is referred to as effectiveness is first important in a workplace. I would like to differ on this point especially on finding out the mentees, how far this will be make changes in workplace burnout? But nevertheless the topic was dealt in a nice manner and well written opinion and need of the hour.

Reviewer Expertise:

NA

No decision status is available

# Reviewer report: Reviewer response for version 1

**DOI:** 10.21956/mep.19976.r30378

**Published:** 2020-02-14

**Authors:** Leila Niemi-Murola

**Affiliations:** 1 University of Helsinki

This review has been migrated. The reviewer awarded 4 stars out of 5 This is an interesting article focusing on a hot topic, burnout among medical students and residents, which condition affects a growing number of our colleagues. There is an urgent need for interventions to assist these young people to cope in their working life as physicians. The authors list ten pieces of good advice and justify them. After all, residency is a short period in ones working life. However, these pieces of advice are culturally sensitive and they are not applicable in all countries. In Nordic counties, it is possible to find a balance between work and family life with young children. There is plenty of literature about root causes of burnout and the mechanism causing it, but not so many professional reflections about the prevention of this painful phenomenon.

Reviewer Expertise:

NA

No decision status is available

# Reviewer report: Reviewer response for version 1

**DOI:** 10.21956/mep.19976.r30376

**Published:** 2020-02-13

**Authors:** Carla Todaro

**Affiliations:** 1 Azienda Sanitaria Universitaria Integrata di Trieste

This review has been migrated. The reviewer awarded 5 stars out of 5 This piece gives food for thought about different career phases and how simple tips can be helpful to manage stressful stages of professional life.The 10 points focus the attention on the importance to do not lose the attitude to be “a continue learner”, be effective in the clinical work but also expert on one unique topic in order to become a referral.But the most important point is the ability to ‘juggle work and life”… Probably the most difficult tip to follow but fundamental point to avoid burnout.Also a mentor can always make a different in a mentee career path…opening new perspective and stimulating reflection...we all should never forget this!Work satisfaction is the key to a fulfilling professional development!Thanks for this paper full of good vibes!!!

Reviewer Expertise:

NA

No decision status is available
